# Attachment styles and sense of coherence as indicators of treatment adherence and completion among individuals with substance use disorder

**DOI:** 10.1186/s13722-025-00578-7

**Published:** 2025-06-12

**Authors:** Nóra Kerekes, Kourosh Bador, Carro Smedeby, Linus Hansen, Sofie Lundström, Monica Eriksson

**Affiliations:** 1 Department of Health Sciences, University West, 461 86 Trollhättan, Sweden; 2 Centre for Holistic Psychiatry Research (CHoPy), 431 60 Mölndal, Sweden; 3 AGERA KBT, 411 38 Gothenburg, Sweden; 4 Department of Psychology, Lund University, 22100 Lund, Sweden

**Keywords:** Attachment styles, Integrated intervention, Sense of coherence (SOC), Substance use disorder (SUD), Sweden, Treatment adherence, Treatment completion

## Abstract

**Background:**

Substance use disorder (SUD) is a growing public health concern in Sweden. Various treatments for SUD exist, with motivational treatment, cognitive behavioral therapy, and relapse prevention being the nationally recommended approaches. Attachment theory and the salutogenic theory with its core concept, sense of coherence (SOC) provides valuable insights into individuals’ available personal resources and their potential for adherence to treatment. The aims of the present study were to examine attachment styles (secure, insecure-avoidant, and insecure-anxious) and SOC (overall and dimensional - comprehensibility, manageability, and meaningfulness- scores) in individuals with SUD; to explore potential correlations between these constructs ; and to assess their predictive value for treatment completion.

**Methods:**

Clinical data were collected between 2014 and 2023 from 164 clients at a Swedish outpatient clinic for addiction who initiated the intensive, integrated treatment program. The sample comprised 109 men and 55 women, aged 18 to 72 years (M = 40.71). Data were gathered using validated self-report instruments (the Attachment Style Questionnaire and the Sense of Coherence Questionnaire). Statistical analyses included descriptive statistics, correlation analysis, and logistic regression.

**Results:**

Individuals with SUD predominantly exhibited an insecure-avoidant attachment style. The four dimensions reflecting insecure attachment (discomfort with relationships, relationships as secondary, need for approval, and preoccupation with relationships) were negatively correlated with overall SOC and its three components.  In contrast the secure attachment dimension (confidence in self and others) showed  positive association with SOC. The strongest associations were found between the manageability component of SOC and all attachment dimensions. The insecure-anxious attachment style showed the strongest association with early dropout from treatment, while higher manageability was significantly associated with  an increased likelihood of treatment completion.

**Conclusion:**

The predominance of an insecure-avoidant attachment style among clients undergoing intensive, integrated treatment for SUD underscores the importance of reinforcing a secure attachment and strengthening SOC to facilitate treatment completion. These findings highlight the need for comprehensive, integrated social and psychiatric care for individuals with SUD.

## Introduction

Attachment theory is a developmental psychological theory that was originally presented in John Bowlby’s classic work *Attachment* (1982). Notably, attachment is not confined to childhood; it plays a role in shaping individuals’ interactions throughout their lives, with early patterns influencing later relationships [1]. This is consistent with findings from modern neurobiological research, which suggest that the brain’s responses associated with attachment styles persist into adulthood [2]. Early patterns of attachment have been shown to influence later relationships, although significant life events, mental health challenges, and therapeutic interventions can modify these patterns [3]. The importance of attachment styles in mental health is well-supported. A recent meta-analysis confirmed that insecure attachment styles are strongly associated with higher levels of anxiety, depression, and other psychological difficulties, while secure attachment is linked to better emotional well-being [4].

Insecure attachment styles are overrepresented in individuals with substance use disorder (SUD) compared to the general population. These attachment patterns are associated with heightened vulnerability to psychological distress and substance use [5, 6]. Research has suggested that insecure attachment styles may serve as vulnerability factors for the development of SUD rather than a consequence of the condition [7].

Additionally, attachment styles may influence treatment engagement and outcomes. Studies have demonstrated that insecure-avoidant attachment is associated with lower motivation for treatment and reduced receptivity to psychotherapy, whereas insecure-anxious attachment is associated with higher motivation but greater emotional reactivity during therapy [8, 9]. Moreover, insecure attachment styles are associated with increased difficulties in therapeutic relationships, which are critical for successful treatment outcomes [10].

Sense of coherence (SOC), a key construct in salutogenic theory, reflects an individual’s ability to perceive life as comprehensible, manageable, and meaningful [11–13]. Strong SOC is associated with better stress management, improved health behaviors, and reduced psychological distress [14, 15]. Conversely, weaker SOC has been linked to poorer treatment outcomes in individuals with SUD, including higher dropout rates and relapse [16–18]. Strengthening SOC through targeted interventions may enhance pro-health behaviors and reduce the risk of relapse in individuals undergoing SUD treatment [14, 19]. SOC may serve as a mediator in the relationship of attachment and mental health. For example, Ying et al. [20] found that individuals with stronger SOC tend to experience less distress in the presence of insecure attachment patterns. This interaction suggests that enhancing SOC could mitigate some of the adverse effects of insecure attachment on treatment adherence and psychological outcomes.

Despite extensive research on attachment and SOC as separate constructs, there is limited understanding of how these two frameworks interact in individuals with SUD. Attachment theory provides insight into relational and emotional vulnerabilities, while SOC offers a lens for understanding how individuals manage stress and adversity. Combining these perspectives may provide a more holistic understanding of the factors influencing treatment adherence and outcomes in individuals with SUD.

This study aimed to examine attachment styles and SOC among individuals with SUD, explore potential correlations between specific attachment styles and SOC dimensions, and assess the predictive value of these  constructs in relation to treatment completion versus early dropout from an intensive, integrated outpatient addiction program. By addressing the gap in understanding the interplay between attachment and SOC in SUD populations, this study contributes to the development of more targeted and effective treatment approaches.

## Material and methods

### Design and intervention

The study analyzed clinical data from clients in a four-month intensive, integrated psychological treatment program for substance use disorder (SUD) at a private outpatient clinic in western Sweden. Referred by social services, clients were informed about the program’s structure and ethical guidelines, and completed a survey on demographics and validated mental health measures before the treatment began. The program was delivered five days a week and combined psychoeducation, cognitive and behavioral therapies, mindfulness, skills training, standardized acupuncture, and structured yoga sessions.

### Study population

Clinical data were collected between 2014 and 2023 from 164 clients (109 men, 55 women) aged 18–72 years (M = 40.71; SD = 11.85). Men and women had similar mean ages (M = 40.95 vs. 40.24). Most clients (73%) were single (74.3% of men, 70.9% of women), and 46% reported having children, averaging one child. Clients were referred by social services and did not undergo formal psychiatric evaluation but completed validated self-report measures of anxiety (Beck Anxiety Inventory; [21]) and depression (Beck Depression Inventory [22]). Mean anxiety scores were 21.96 (20.76 for men, 24.37 for women), and depression scores averaged 24.16 (23.27 for men, 25.91 for women), indicating moderate symptoms in both domains, according to Beck’s criteria.

### Data collection

**Fig. 1:**
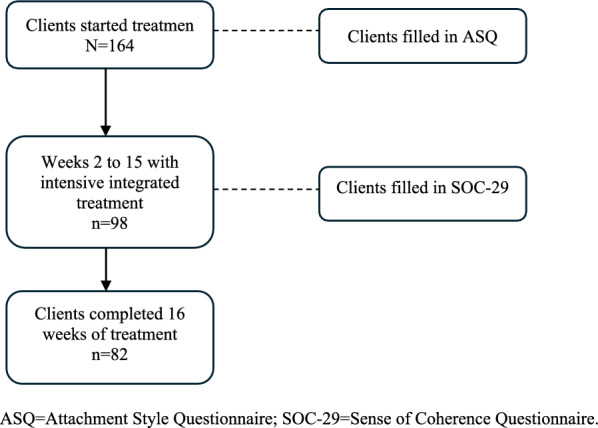
Clients’ participation in intensive, integrated treatment over time

All 164 clients who consented to participate in the standardized, integrated, intensive addiction treatment program conducted a self-assessment of their attachment styles, using the Attachment Style Questionnaire (ASQ), as part of a clinical admission survey. The Sense of Coherence Questionnaire (SOC-29) was used to measure the strength of the clients’ SOC. As there was a continuous intake to the intervention, clients entered the treatment structure at different points; therefore, they completed the SOC-29 at various times during the intervention. An overview of the clients’ participation is shown in Fig. 1.

### Instruments

#### Attachment style questionnaire (ASQ)

**Fig. 2:**
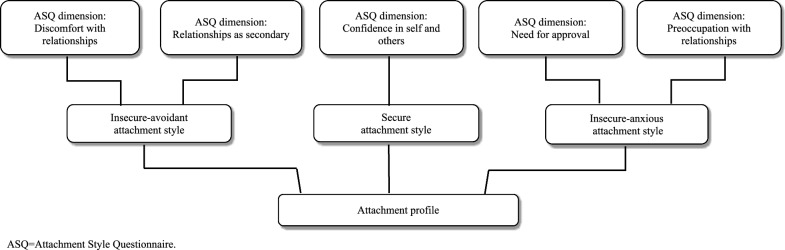
The dimensions of the ASQ and how they correspond with attachment styles

The Attachment Style Questionnaire (ASQ) is a 40-item self-report instrument based on attachment theory, originally developed in English [23] and later translated into Swedish [24]. It assesses individuals’ attitudes toward themselves and others using a six-point Likert scale ranging from “totally disagree” to “totally agree.” The questionnaire identifies attachment styles across five dimensions: confidence in self and others, discomfort with relationships, relationships as secondary, need for approval, and preoccupation with relationships. Confidence in self and others represents a secure attachment style, characterized by trust and comfort in relationships, while the remaining four dimensions reflect aspects of insecure attachment. Discomfort with relationships and relationships as secondary are linked to insecure-avoidant patterns and concern attitudes toward others. High scores on these dimensions often indicate discomfort with intimacy or a preference for independence. Need for approval and preoccupation with relationships relate to insecure-anxious attachment and focus on self-perception, involving excessive reassurance-seeking and dependency for a sense of belonging [23]. An overview of the dimensions of the ASQ and how they are connected to attachment styles is presented in Fig. 2.

In this study, ASQ dimension scores were compared with values from a Swedish reference population of 90 individuals aged 18 to 42 [24]. Mean scores were calculated for each dimension. A secure attachment classification required a score above 4.0 on the secure dimension, combined with insecure scores at or below reference levels. If the insecure dimensions exceeded reference values, the profile was considered predominantly insecure, although a high score in the secure dimension could indicate moderating effects.

The Swedish version of the ASQ has demonstrated acceptable internal consistency, with Cronbach’s alpha values between 0.71 and 0.84 [24]. In clinical samples, reliability has been somewhat lower. In particular, the relationships as secondary dimension showed poor reliability (α = 0.56) in a prior study [25], and was also the lowest in the current study (α = 0.67). The other dimensions showed acceptable reliability: discomfort with relationships (0.81), confidence in self and others (0.83), need for approval (0.74), and preoccupation with relationships (0.77).

#### Sense of coherence questionnaire (SOC-29)

The SOC-29, also known as the Orientation to Life Questionnaire, is a self-assessment tool that assesses an individual’s perception of comprehensibility, manageability, and meaningfulness. It contains 29 questions (11 that focus on comprehensibility, 10 on manageability, and 8 on meaningfulness) that are each answered using a 7-point Likert scale. The total score ranges from 29 to 203 points [26]. The cutoff point is not standardized and should be contextually linked to the research question. The total scale has consistently shown a high level of internal consistency, with α values ranging from 0.84 to 0.93 across several studies [12]. In the present study, the α for the SOC-29 was 0.89, indicating high internal reliability. The internal reliability of each individual SOC dimension was acceptable (comprehensibility, α = 0.69; manageability, α = 0.73; meaningfulness, α = 0.84).

### Statistical analysis

All data were analyzed using SPSS (version 29.0.2.0, IBM). Normality was assessed with the Shapiro–Wilk test. Due to deviations in certain ASQ and SOC-29 dimensions, non-parametric tests were subsequently applied. Descriptive statistics (means, standard deviations, and percentages) were reported, with significance set at *p* < 0.05. Relationships between attachment styles and SOC dimensions were examined using Spearman correlations, interpreted as follows: r < 0.1 (no association), 0.1–0.3 (weak), 0.3–0.6 (moderate), and > 0.6 (strong) [27]. Logistic regression was conducted to examine early dropout using attachment as a predictor. A second regression assessed completion of the intensive intervention, using both attachment and SOC scores as predictors. SOC-29 was excluded from the dropout model, as it had not been completed at that intervention stage.

## Results

### Attachment styles and SOC scores

**Fig. 3:**
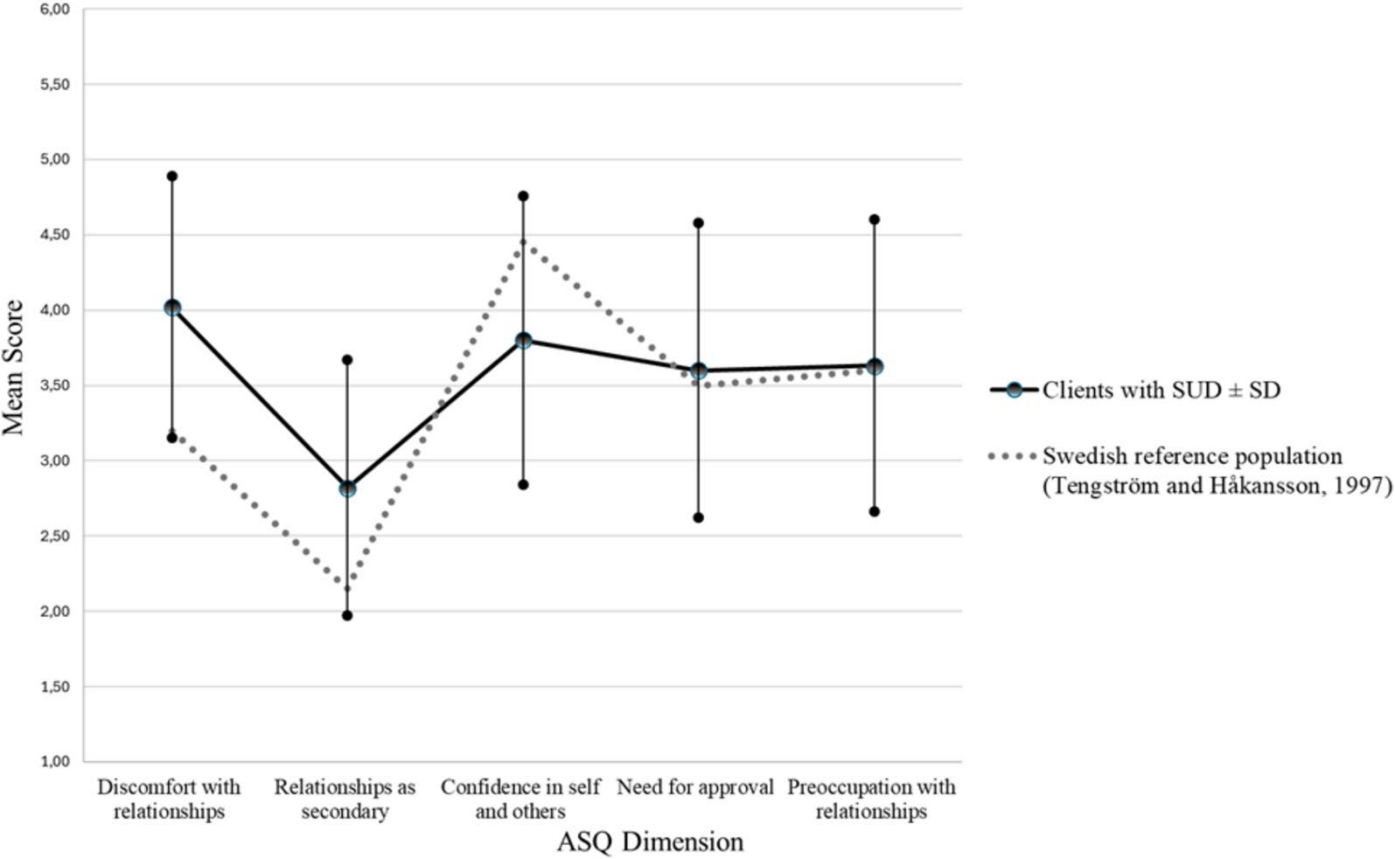
Attachment profiles of the study and reference populations

Descriptive statistics for the study population with regard to the dimensions of the ASQ and SOC-29 are presented in Tables 1 and 2. A visual representation of the clients’ attachment profiles is shown in Fig. 3.

**Table 1:** Attachment styles and ASQ dimension scores among the study population

Attachment style	ASQ dimension	M (SD)	Md	Min–Max
Insecure-avoidant	Discomfort with relationships	4.02 (0.87)	4.10	2.10–5.90
	Relationships as secondary	2.82 (0.85)	2.71	1.00–5.29
Secure	Confidence in self and others	3.80 (0.96)	3.88	1.25–5.75
Insecure-anxious	Need for approval	3.60 (0.98)	3.71	1.43–6.00
	Preoccupation with relationships	3.63 (0.97)	3.56	1.25–5.75

ASQ = Attachment Style Questionnaire; M = mean; SD = standard deviation; Md = median

**Table 2:** SOC-29 scores among the study population

SOC-29 Dimension	M (SD)	M_A_	Md	Min–Max
Total SOC-29 score	121.07 (21.49)	4.17	118.50	77–172
Meaningfulness (8 items)	37.55 (7.87)	4.69	37.00	20–54
Comprehensibility (11 items)	40.90 (7.85)	3.71	40.00	23–55
Manageability (10 items)	42.62 (8.55)	4.26	42.50	25–64

SOC-29 = Sense of Coherence Questionnaire; M = mean; SD = standard deviation; M_A_ = mean adjusted for the number of items within the dimension; Md = median

The clients exhibited moderate mean scores for four of the five dimensions of the ASQ (Table 1, Fig. 3). These dimensions included “confidence in self and others,” “discomfort in relationships,” “need for approval,” and “preoccupation with relationships.” The “relationships as secondary” dimension exhibited the lowest mean score.

The clients in the present study indicated a higher prevalence of insecure-avoidant attachments than the reference population. In the present study, the mean scores for “discomfort with relationships” and “relationships as secondary” were 0.8 and 0.67 points higher than the reference values, respectively. The present study population exhibited a lower prevalence of secure attachments than the reference population, with a mean score 0.65 points lower than the reference value. The prevalence of insecure-anxious attachments was slightly higher in the present study population than in the reference population, with differences of 0.10 and 0.03 points for the “need for approval” and “preoccupation with relationships” dimensions, respectively.

The clients’ mean SOC-29 score was 121. When adjusted for the number of items included in each dimension, the corrected mean scores were comparable across all dimensions. The highest score for the present study population corresponded with meaningfulness (M_A_ = 4.69), and the lowest corresponded with comprehensibility (M_A_ = 3.71) (Table 2).

### Associations between attachment styles and SOC dimensions

The correlations between attachment styles and SOC dimensions are presented in Table 3.

**Table 3:** Strength and significance of associations between attachment styles and SOC

		SOC dimension							
		Total SOC		Meaningfulness		Comprehensibility		Manageability	
Attachment Style	ASQ Dimension	r	*p*	r	*p*	r	*p*	r	*p*
Insecure-avoidant	Discomfort with relationships	− 0.38	< 0.001	− 0.40	< 0.001	− 0.24	0.019	− 0.36	< 0.001
	Relationships as secondary	− 0.33	< 0.001	− 0.33	< 0.001	− 0.18	0.08	− 0.34	< 0.001
Secure	Confidence in self and others	0.43	< 0.001	0.47	< 0.001	0.23	0.022	0.41	< 0.001
Insecure-anxious	Need for approval	− 0.40	< 0.001	− 0.29	0.004	− 0.27	0.006	− 0.44	< 0.001
	Preoccupation with relationships	− 0.33	< 0.001	− 0.19	0.06	− 0.21	0.035	− 0.41	< 0.001

ASQ = Attachment Style Questionnaire; SOC = sense of coherence; r = Spearman’s rho; *p* = significance level

A secure attachment style was positively associated with total SOC and all three SOC dimensions, with significant and moderately strong associations between a secure attachment and total SOC, meaningfulness, and manageability. The association between a secure attachment style and comprehensibility was weak. Conversely, insecure attachment styles were negatively associated with total SOC and each of its dimensions. Generally, total SOC and manageability had the strongest associations with attachment style, demonstrating a moderate effect size with each of the attachment styles. The dimension of comprehensibility exhibited the weakest associations; its association with “relationships as secondary” did not reach statistical significance.

### The predictive capacity of attachment styles and SOC for early dropout and treatment completion

Due to the high dropout rate observed during treatment (40%), a logistic regression analysis was conducted to investigate whether any of the dimensions of the ASQ could predict early dropout. While none of the dimensions showed a statistically significant association with early dropout, “preoccupation with relationships” demonstrated a trend that neared significance (*p* = 0.07). Every 1-point increase in the “preoccupation with relationships” dimension was coupled with a 47% increase in the likelihood of a client dropping out during the course of the intervention (Exp[B] = 1.470).

In terms of predictive capacity for fully completing the intervention, no attachment style was found to have a significant predictive value. Similarly, the SOC dimensions of meaningfulness and comprehensibility did not demonstrate any significant predictive value. However, for each 1-point increase in manageability, there was a significant (*p* = 0.048) increase of approximately 10% in the likelihood of a client completing the intervention (Exp[B] = 1.097).

### Discussion

The clients in the present study predominantly exhibited an insecure-avoidant attachment style. This was particularly clear from the dimensions that assessed the need for interpersonal distance. While a minor elevation was also observed in one of the two dimensions associated with an insecure-anxious attachment, this deviation was minimal in comparison to the scores of the reference population [24]. This suggests that it is specifically an insecure-avoidant attachment style, not a general insecure attachment style, that is representative of the study population. However, the slight increase in insecure-anxious scores may support an overall insecure pattern, particularly when combined with the significantly elevated scores for the dimensions associated with an insecure-avoidant attachment. The insecure attachment style of the clients in the present study was further reinforced by their lower scores in the secure attachment style dimension in comparison with the reference population [24].

A higher prevalence of insecure attachments among individuals with SUD has been previously described [28]. Individuals with an insecure-avoidant attachment style often prefer to maintain distance from others and adopt coping approaches that depend less on other people [24]. They tend to avoid close relationships, which may serve as a protective mechanism against perceived vulnerability in relationships. As a result of adopting a generally distant and avoidant approach toward others, it may be reasonable to assume that individuals with SUD struggle to invest trust in care settings that rely on interpersonal relationships, including therapeutic relationships in the treatment of SUD, other forms of psychiatric care, and general healthcare. Considering that an insecure attachment may be a vulnerability factor for SUD and that SUD poses an elevated risk for comorbidities, attachment style potentially affects both the risk of developing SUD and one’s ability to engage in treatment [7, 29, 30]. Although the present study did not assess health-related treatment outcomes, its confirmation of a predominantly insecure attachment style among clients with SUD underscores the potential benefit of incorporating considerations of attachment style in the treatment of SUD.

#### Associations between attachment styles and SOC dimensions

Generalized Resistance Resources (GRRs) and Specific Resistance Resources (SRRs) are fundamental constructs in the salutogenic theory, which emphasizes the promotion of well-being rather than solely focusing on disease treatment [11]. GRRs are broad, wide-ranging factors such as material resources, ego identity, and social support, which contribute to the development of a strong SOC. While GRRs are typically considered more generic, they can also manifest on an individual level, reflecting a person’s broader capabilities and resources that are not tied to a specific context.

In contrast, SRRs are more individualized and context-dependent, tailored to the person’s immediate needs and specific situations. In the context of clinical SUD treatment programs, SRRs might include a client’s particular coping mechanisms, therapeutic alliances, or access to program-specific resources. These resources enable the individual to effectively manage stressors within the unique environment of the treatment setting.

The salutogenic perspective considers the whole individual—physical, mental, emotional, and existential aspects—aligning with holistic approaches to care. By recognizing the interplay between GRRs and SRRs, care providers can better support individuals by leveraging both generalized and context-specific resources to enhance SOC, thereby promoting resilience and well-being during treatment.

The present study indicates that a secure attachment is positively associated, and insecure attachment styles are negatively associated, with overall SOC and all three SOC dimensions. A secure attachment style, characterized by confidence in oneself and others, can be considered a GRR. This is in line with the salutogenic theory and the definition of SOC—“…the extent to which one has a pervasive, enduring though dynamic feeling of confidence…” [11], *p*. 19). Individuals with secure attachments view themselves and others positively, facilitating better stress management and coping strategies. Overall, a secure attachment enhances both GRRs and SRRs and may act as an SRR in particular situations providing emotional support and fostering strong interpersonal relationships.

Previous research has highlighted the role of attachment styles in shaping SOC [31], which, in turn, may mediate the impact of an insecure attachment on subjective well-being [32]. Particularly notable in the context of adult clients with SUD are the significant and moderately strong associations between attachment styles and manageability, as shown in the present study. This suggests that manageability has the most pronounced influence on perceived health of the three dimensions of SOC. Previous studies have demonstrated that a more secure attachment style and a stronger SOC might have potential benefits for health and well-being [14, 29]; the current findings highlight the potential advantages of integrating interventions aimed at enhancing secure attachments and SOC into the treatment of SUD. The maintenance of good health requires the ability to effectively manage stressful situations [11]. Therefore, supporting individuals with SUD in strengthening their sense of manageability, helping them identify and utilize both personal and formal resources, is crucial. This support enables these individuals to effectively manage their situations and improve their health.

Moreover, SOC has been identified as a significant and central aspect of recovery capital, which encompasses both internal and external resources that support long-term recovery from SUD, including cultural, physical, human, and social capital. A moderately-to-strongly positive correlation between recovery capital and SOC suggests that strengthening SOC can equip individuals with additional resources to help them manage situations related to substance use [33]. By adapting a salutogenic perspective, individuals with SUD can develop a stronger SOC through coping strategies for better perceived health. Previous research has shown an association between improved SOC and better subjective health, including mental health, physical health, quality of life, and well-being [15, 34],this reinforces the positive effects of integrating a salutogenic perspective for clients undergoing interventions. From a care science perspective, an understanding of the possible impacts of attachment style and SOC on an individual’s preconditions for treatment and health has the potential to provide psychiatric and health care professionals with valuable insights into the individual’s coping mechanisms, thereby improving the conditions for tailored and person-centered care. This underscores the potential benefits of incorporating elements from these frameworks into interventions for SUD and future research to further explore their interconnectedness [17, 19].

### Prediction of early dropout and treatment completion

During the four-month, intensive, integrated treatment intervention for SUD, a substantial portion of clients opted to discontinue the program; 40% ceased treatment before completing the SOC-29 questionnaire. The observed correlation between early dropout and a predominantly insecure-anxious attachment style, notably within the “preoccupation with relationships” dimension, suggests that individuals who tend to excessively seek connection with others may not find this type of intervention suitable. However, it is important to interpret this result with some caution, as the correlation approached but did not reach significance (*p* = 0.07). While we cannot definitively explain why an insecure-anxious attachment was linked with early dropout from the intervention, a potential explanation may lie in the characteristic traits of this attachment style. Specifically, the dimension “preoccupation with relationships,” as described by Tengström and Håkansson [24], is characterized by an exaggerated fixation on relationships, where individuals seek intense contact to fulfill their needs for security in interpersonal connections. Given the intensity of the integrated treatment program, which involved active interventions five days per week [35], certain aspects of this format may have been overwhelming for these clients. Additionally, this group may have been particularly sensitive to staff turnover or other group-specific dynamics.

The link between attachment insecurity and challenges in SUD treatment is well-supported in the literature. Fletcher and Nutton [36] emphasized that individuals with insecure attachment styles often struggle with emotional regulation and interpersonal trust, which can adversely affect their engagement in treatment. Similarly, Cihan et al. [37] discussed the etiological role of attachment insecurity in the development and maintenance of substance use disorders. According to their work, insecure attachment patterns, including anxious-preoccupied attachment, may increase susceptibility to maladaptive coping mechanisms like substance use, particularly in the context of unmet relational needs.

Recent studies further support these observations. For example, Rübig et al. [38] demonstrated that insecure attachment patterns are highly prevalent among SUD patients and are associated with weaker therapeutic alliances and reduced therapy motivation, both of which are critical for treatment retention. These findings suggest that the insecure-anxious attachment style observed in our study may have hindered clients’ ability to engage effectively with the treatment program, contributing to early dropout. Similarly, Vismara et al. [39] found that preoccupied attachment styles, which are characterized by heightened dependency and emotional dysregulation, were more common among SUD outpatients compared to individuals in therapeutic communities, further highlighting the interplay between attachment patterns and treatment preferences or suitability. Interestingly, Fuchshuber et al. [40] noted that while attachment security is generally associated with positive treatment outcomes, it may paradoxically relate to early dropout in some contexts. This finding underscores the complexity of attachment dynamics in treatment settings and the need to consider individual differences in treatment planning.

Notably, treatment completion was significantly associated with the SOC dimension of manageability. This suggests that the intervention may have been demanding and stressful in terms of individuals’ abilities to manage their situations, since a higher manageability score was associated with an increased likelihood of a client completing the entire four-month intervention. Specifically, each additional point in the manageability dimension was associated with a 10% increase in the likelihood of treatment completion.

## Strengths and limitations

This study benefits from several methodological strengths. Clinical data were collected from a substantial sample of 164 individuals over a nine-year period (2014–2023), enhancing both representativeness and reliability. The use of validated instruments—ASQ and SOC-29—known for their strong internal consistency, further strengthens the credibility of the findings. In addition, systematic procedures in data collection and analysis support the study’s internal validity.

However, certain limitations must be acknowledged. The sample showed a gender imbalance (109 men, 55 women), which may affect generalizability. Still, existing literature indicates that neither attachment styles nor SOC are inherently gender-dependent constructs [11, 41]. The study also lacked data on key social determinants of health—such as education, income, housing, and employment—which could influence treatment outcomes. Including these variables in future research could provide a more nuanced understanding of the role of socioeconomic factors.

Timing of the SOC-29 administration varied, potentially introducing bias, as participants may have been influenced by their stage in treatment. Moreover, the absence of pre- and post-intervention measures limited the ability to assess change over time. The “relationships as secondary” dimension of the ASQ demonstrated suboptimal reliability (α < 0.70), consistent with previous findings, suggesting it may require revision or contextual adaptation. Lastly, the lack of definitive cutoff scores for SOC-29 complicates interpretation, warranting cautious conclusions regarding the strength of participants’ sense of coherence.

## Conclusions

Clients with SUD who underwent intensive outpatient treatment in Sweden predominantly displayed insecure attachment patterns, especially of the avoidant type. This aligns with previous research and highlights the interpersonal difficulties such clients may face in therapeutic settings, where emotional closeness and trust are essential. The findings suggest that attachment styles can significantly influence both treatment processes and outcomes.

The study also confirmed associations between attachment styles and the SOC framework, particularly with overall SOC and the dimension of manageability. These results build on existing evidence and support the idea that addressing attachment-related issues may strengthen SOC and, in turn, improve treatment efficacy.

Moreover, the data revealed that an insecure-anxious attachment style was linked to early treatment dropout, while higher manageability scores were strongly associated with treatment completion. Given the high attrition rates often seen in SUD treatment, these findings underscore the need for novel approaches that promote secure attachment and enhance clients’ coping capacity to support treatment adherence and long-term recovery.

## Recommendations for practical application

The results emphasize the importance of incorporating attachment theory into SUD treatment. The high prevalence of insecure, particularly avoidant, attachment styles suggests a need for relational approaches that prioritize emotional safety and trust. Understanding individual attachment profiles within integrated interventions can inform personalized treatment strategies. Even in the absence of detailed assessments, clinicians' awareness of common attachment difficulties can help foster therapeutic alliances and promote engagement.

In addition, the strong link between manageability and treatment retention points to the value of interventions that build clients’ coping resources. Enhancing psychoeducation and social support may reinforce resilience, reduce dropout, and improve treatment outcomes for individuals with SUD.

## Data Availability

The data that support the findings of this study are available from KB, but restrictions apply to the availability of these data, which were used under license for the current study, and so are not publicly available. Data are however available from the authors upon reasonable request and with permission of KB.
